# Potential Utility of Urinary Follistatin as a Non-Invasive Indicator of Acute Tubular Damage in Patients with Acute Kidney Injury

**DOI:** 10.3390/cells13060525

**Published:** 2024-03-16

**Authors:** Izumi Nagayama, Kaori Takayanagi, Daisuke Nagata, Hajime Hasegawa, Akito Maeshima

**Affiliations:** 1 Department of Nephrology and Hypertension, Saitama Medical Center, Saitama Medical University, Kawagoe 350-8550, Japan; 2 Division of Nephrology, Department of Internal Medicine, Jichi Medical University, Shimotsuke 329-0431, Japan

**Keywords:** follistatin, acute kidney injury, urinary biomarker

## Abstract

Activin A is known to impede tubular repair following renal ischemia, whereas exogenous follistatin, an activin A antagonist, has been shown to ameliorate kidney damage in rats. Despite these findings, the precise role of endogenous follistatin in the kidney has yet to be elucidated. In this study, we investigated the localization of follistatin in the normal human kidney and its potential utility as a marker for acute kidney injury (AKI). In a total of 118 AKI patients and 16 healthy adults, follistatin levels in serum and urine were quantified using ELISA, and correlations with clinical parameters were analyzed. Follistatin-producing cells were positive for Na-Cl co-transporter and uromodulin, but negative for aquaporin 1 and aquaporin 2. Unlike healthy adults, urinary follistatin significantly increased in AKI patients, correlating positively with AKI severity. Urinary follistatin levels were notably higher in patients needing renal replacement therapy. Significant correlations were observed with urinary protein, α1 microglobulin, and urinary NGAL, but not with urinary KIM-1, urinary L-FABP, urinary NAG, urinary β2 microglobulin, or serum creatinine. Interestingly, no correlation between urinary and serum follistatin levels was identified, indicating a renal origin for urinary follistatin. In conclusion, follistatin, produced by distal tubules, is detectable in the urine of AKI patients, suggesting its potential as a valuable marker for monitoring acute tubular damage severity in AKI.

## 1. Introduction

Acute kidney injury (AKI) is a prevalent yet intricate medical condition that is linked to increased morbidity and mortality [1,2]. AKI is often associated with adverse outcomes if not promptly identified and managed. Factors that contribute to AKI include renal ischemia, sepsis, and nephrotoxic drugs, and the global annual incidence is rising. AKI poses a substantial risk for the development of chronic kidney disease (CKD) [3,4,5,6,7]. Even in mild instances, AKI adversely impacts not only renal but also overall prognosis [8,9]. Early recognition of AKI is crucial for initiating interventions to minimize renal damage and improve patient outcomes. Timely diagnosis allows for the implementation of targeted therapeutic strategies, such as fluid management, medication adjustments, and, in severe cases, renal replacement therapy. The current diagnostic criteria for AKI, which are reliant on serum creatinine levels and urine output, may exhibit limited sensitivity, leading to delayed intervention.

Traditionally, AKI diagnosis has relied on an increase in serum creatinine levels, yet its sensitivity and specificity for detecting acute declines in renal function are suboptimal. To address this challenge, several AKI biomarkers, including NGAL [10], KIM-1 [11], IL-18 [12], L-FABP [13], and TIMP-2*IGFBP-7 [14,15] have been identified as predictors of the onset of AKI, the severity of AKI, and renal prognosis of AKI in critically ill patients. However, the therapeutic options for AKI are limited, and suitable intervention biomarkers remain elusive.

Follistatin, a single-chain glycoprotein hormone expressed in various tissues, including the ovary, testis, uterus, liver, brain, and kidney, has garnered attention. Recognized for its ability to suppress follicle-stimulating hormone secretion, follistatin possesses distinct properties from inhibin [16] and functions as an endogenous antagonist of activin A [17], a multifunctional cytokine belonging to the transforming growth factor-β (TGF-β) superfamily, which regulates cell growth and differentiation in various organs. Activin A has been shown to negatively regulate tubular regeneration after renal ischemia [18]. Expression of mRNA for activin A was absent in normal kidneys but significantly increased after renal ischemia. Activin protein was found in tubular cells of the outer medulla in ischemic but not normal kidneys. Exogenous administration of recombinant follistatin prevented the histologic changes induced by renal ischemia, reduced apoptosis in tubular cells, and promoted tubular cell proliferation. Serum levels of creatinine and blood urea nitrogen were significantly lower in follistatin-treated rats [18]. The upregulation of activin A in tubular cells after renal ischemia in rats further suggests its role as a paracrine factor activating renal interstitial fibroblasts during kidney fibrotic processes [19]. Immunoreactive activin A was upregulated in tubular cells in the kidneys with unilateral ureteral obstruction, but not in normal and contralateral kidneys. Activin A induced cell growth, enhanced the expression of type I collagen, and induced the production of alpha-smooth muscle actin in a rat kidney fibroblast cell line (NRK-49F cells) as well as in primary cultured renal interstitial fibroblasts. Follistatin significantly reduced cell growth in NRK-49F cells. Activin A expression was induced by TGF-beta 1 or activin A itself. Induction of type I collagen expression by TGF-beta 1 was reduced by follistatin [19]. In addition, exogenous follistatin administration has demonstrated a reduction in renal damage in models of cisplatin nephropathy [20] and CKD models [21].

Although previous studies have reported that urinary activin is detected in mouse models with ischemia-reperfusion injury [22] and human AKI [23], correlating with renal injury severity, whether urinary follistatin is present in AKI patients remains uncertain. The present study aimed to elucidate the localization of follistatin in the normal human kidney and quantify urinary follistatin levels in AKI patients, thereby investigating the potential of urinary follistatin as a marker for AKI.

## 2. Materials and Methods

### 2.1. Setting and Patients

One hundred eighteen patients with AKI treated at Jichi Medical University Hospital and Saitama Medical Center from December 2018 to January 2023 were included in the present study. AKI was diagnosed and staged for severity according to the Kidney Disease Improving Global Outcomes (KDIGO) guidelines [24]. Patients with an estimated GFR < 45 mL/min/1.73 m^2^ before the onset of AKI and patients with anti-neutrophil cytoplasmic antibody-associated vasculitis were excluded. Serum and urine were obtained from living kidney donors before nephrectomy (n = 16), serving as healthy controls (HC). The unaffected parts of nephrectomized kidneys from patients with renal cell carcinoma were used as normal human kidney. The protocol of this study was approved by the ethics committee on human research of Jichi Medical University (approval number A18-081, A18-089, A18-213) and Saitama Medical Center, Saitama Medical University (approval number 2487). All experiments were conducted in accordance with the relevant guidelines and regulations. Written informed consent for study participation was obtained from each patient and control subject. 

### 2.2. Sample and Data Collection

Urine and serum samples were obtained from AKI patients at the time of diagnosis and stored at −80 °C until analysis. In certain instances, urine and serum were collected sequentially until discharge. Clinical data at diagnosis, such as age, gender, complete blood count, serum and urine biochemical parameters, medications, complications, duration of renal replacement therapy (RRT), survival, and cause of death, were extracted from the medical records of each patient.

### 2.3. Immunohistochemical Analysis

Immunostaining was performed using the DAKO EnVision system (DAKO EnVision labelled polymer, peroxidase) as described previously [25]. Briefly, paraffin-embedded sections (3 μm thick) were deparaffinized and hydrated using standard methods. The sections were then treated with a protein block serum-free solution (Dako, Santa Clara, CA, USA) and incubated overnight at 4 °C with the primary antibody. After washing with Tris-buffered saline containing 0.1% Tween 20 (TBS-T), the sections were incubated with a peroxidase-conjugated secondary antibody, followed by visualization with diaminobenzidine and counterstaining with hematoxylin. Subsequently, the slides were examined and photographed under a light microscope BX-61 (Olympus Optical Co., Ltd., Tokyo, Japan), and the images were analyzed. Indirect fluorescent immunostaining was performed as follows. Briefly, sections were incubated with fluorescein-labeled secondary antibodies (Alexa; Molecular Probes, Eugene, OR, USA) and 4′,6′-diamidino-2′-phenylindole dihydrochloride (DAPI). Fluorescent images were recorded with the BX-61 fluorescence microscope (Olympus Optical Co., Ltd., Tokyo, Japan). For the immunostaining control, the primary antibody was replaced with PBS, which did not show positive staining, confirming specificity. Primary antibodies used in the present study were as follows: rabbit anti-follistatin antibody (1:200; LS-B 14665; Lifespan Biosciences, Seattle, WA, USA), mouse anti-aquaporin 1 antibody (1:2500; sc-25287; Santa Cruz Biotechnology, Santa Cruz, CA, USA), mouse anti-aquaporin 2 antibody (1:2500; sc-515770; Santa Cruz Biotechnology), rabbit anti-uromodulin antibody (1:7500; HPA043420; Sigma-Aldrich, St. Louis, MO, USA), and rabbit anti-NCC antibody (1:7500; ab3553; Abcam, Cambridge, UK).

### 2.4. ELISA

Urinary and serum human follistatin (DY669), urinary human KIM-1 (kidney injury molecule-1; DY1750B), urinary human NGAL (neutrophil gelatinase-associated lipocalin; DY1757), and urinary human L-FABP (liver-type fatty acid-binding protein; DY9465) (R&D Systems, Minneapolis, MN, USA) were measured by ELISA according to the manufacturer’s instructions.

### 2.5. Statistical Analysis

All statistical analyses were conducted using GraphPad Prism 8.3 (GraphPad Software Inc., San Diego, CA, USA). Two-group comparisons were performed using a two-sided *t*-test for normally distributed data and the Mann–Whitney test or Wilcoxon test for skewed data. For comparisons involving more than two variables, the Kruskal–Wallis test was used, followed by Dunn’s multiple comparison test to adjust the probability. Correlations were assessed using Spearman’s rank correlation coefficient. Normality was evaluated using the Shapiro–Wilk test. Results with a *p*-value less than 0.05 were considered statistically significant.

## 3. Results

### 3.1. Localization of Follistatin in Normal Human Kidney

**Figure 1:**
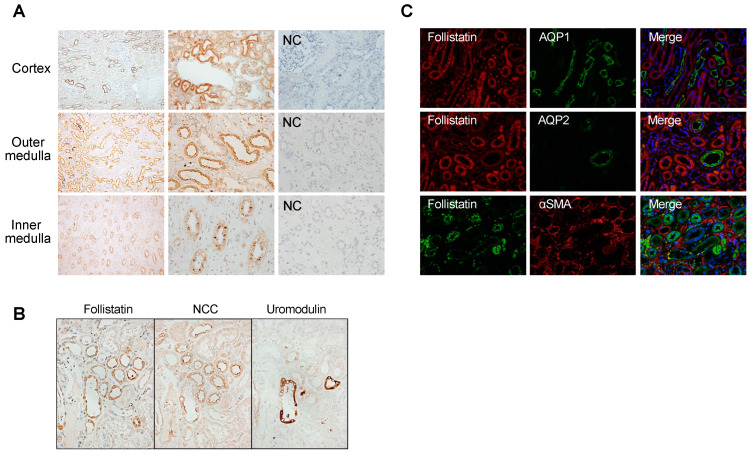
Localization of follistatin in normal human kidney.(**A**) Localization of follistatin in normal human kidney was examined by immunostaining. Follistatin (brown). NC, negative control. Magnification: ×100 (left panels) and ×400 (middle and right panels). (**B**) Identification of follistatin-positive tubules in normal human kidney using serial sections. Nonselective cation channels: NCC. Magnification: ×400. (**C**) Double staining of follistatin with aquaporin 1 (AQP1), aquaporin 2 (AQP2), or α-SMA in normal human kidney. Nucleus (blue). Magnification: ×400.

Immunostaining analyses of nephrectomized kidney specimens were conducted to elucidate the subcellular localization of follistatin protein in the normal human kidney. Follistatin was discerned within the renal tubules located in the cortex, outer medulla, and inner medulla (Figure 1A). Subsequent immunostaining using serial sections revealed that follistatin-positive tubules exhibited positivity for uromodulin and NCC (Figure 1B), while concurrently displaying negativity for aquaporin 1 and aquaporin 2 (Figure 1C). These observations suggest that follistatin is synthesized by distal tubules in the normal human kidney.

### 3.2. Baseline Characteristics of Patients

The baseline characteristics of the participants, including both patients and healthy controls, involved in this study are shown in Table 1. Among the 118 patients diagnosed with AKI, 80 were male, and the mean age was 62.2 ± 16.6 years (± standard error). Notably, no statistically significant differences between AKI patients and their healthy counterparts were observed in the prevalence of comorbidities, except for dyslipidemia. In comparison to the healthy controls, AKI patients exhibited significantly elevated levels of blood urea nitrogen (BUN), serum creatinine (Cr), and C-reactive protein (CRP). Conversely, AKI patients demonstrated significantly lower serum sodium (Na), estimated glomerular filtration rate (GFR), and hemoglobin.

### 3.3. Significant Increase in Follistatin in the Urine of AKI Patients

**Figure 2:**
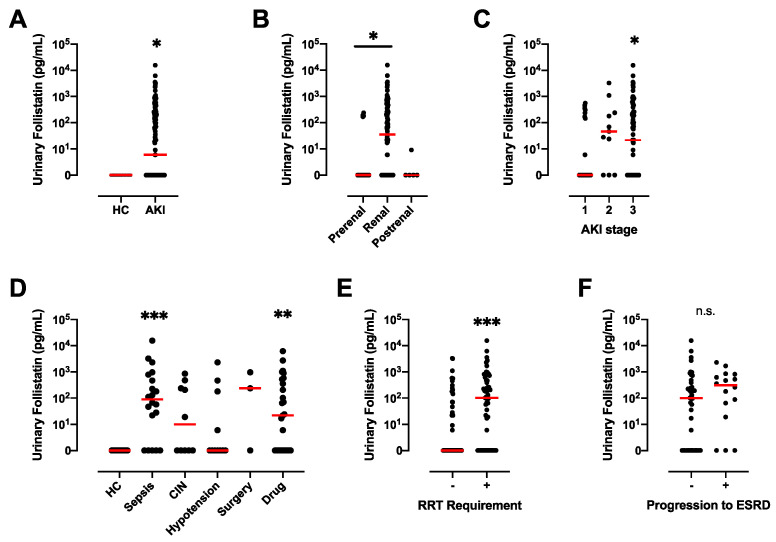
Urinary Follistatin Levels in AKI Patients. (**A**) Urinary follistatin levels in healthy controls (HC) (n = 16) and AKI patients (n = 118). * *p* < 0.05 vs. HC. (**B**) Urinary follistatin levels in patients with prerenal AKI (n = 18), renal AKI (n = 88), and postrenal AKI patients (n = 5). * *p* < 0.05 vs. prerenal AKI. (**C**) Urinary follistatin levels and severity of AKI according to KDIGO stage (stages 1–3). * *p* < 0.05. (**D**) Urinary follistatin levels for each cause of AKI (sepsis, 20; CIN, 10; hypotension, 11; drug-induced, 27; surgery, 3). CIN, contrast-induced nephropathy, N.D., not detected. ** *p* < 0.01, *** *p* < 0.001 vs. HC. (**E**) Urinary follistatin levels in AKI patients who did (+; n = 58) and did not (-; n = 60) require renal replacement therapy (RRT). *** *p* < 0.001 vs. RRT (-). (**F**) Urinary follistatin levels in AKI patients with (+; n = 16) and without (-; n = 42) progression to irreversible ESRD. n.s., not significant. The red line indicates the median.

In the present study, ELISA was used to assess the concentration of follistatin in the urine of patients diagnosed with AKI. The analysis revealed the absence of detectable levels of urinary follistatin in the samples obtained from healthy controls. Conversely, a notable elevation in urinary follistatin was observed in individuals with AKI (Figure 2A). Notably, a statistically significant variance in urinary follistatin levels was discerned between patients with renal AKI and those with prerenal AKI (Figure 2B). Furthermore, when compared to the levels in healthy controls, a substantial increase in urinary follistatin was evident in cases of sepsis-associated AKI (n = 20, 1214 ± 783.4) and drug-induced nephropathy (n = 27, 514.9 ± 246.4), while no such elevation was observed in AKI attributed to hypotension (n = 11, 268.6 ± 207.8), CIN (n = 10, 179.6 ± 90.6) or surgery (n = 3, 402.1 ± 291.7) (Figure 2D).

### 3.4. Urinary Follistatin Is Associated with the Severity and Prognosis of AKI

Next, the association between urinary follistatin levels and the severity of AKI was investigated. A marked elevation in urinary follistatin was observed in AKI stage 3 when compared to stage 1 (Figure 2C). Additionally, we assessed the variation in urinary follistatin levels between individuals who required renal replacement therapy (RRT) and those who did not. Notably, urinary follistatin levels were significantly elevated in patients requiring RRT compared to AKI patients who did not require such an intervention (Figure 2E). Conversely, no statistically significant difference was identified between patients who progressed to irreversible end-stage renal disease (ESRD) and those who did not (Figure 2F).

### 3.5. Correlation between Urinary Follistatin and Clinical Parameters

**Figure 3:**
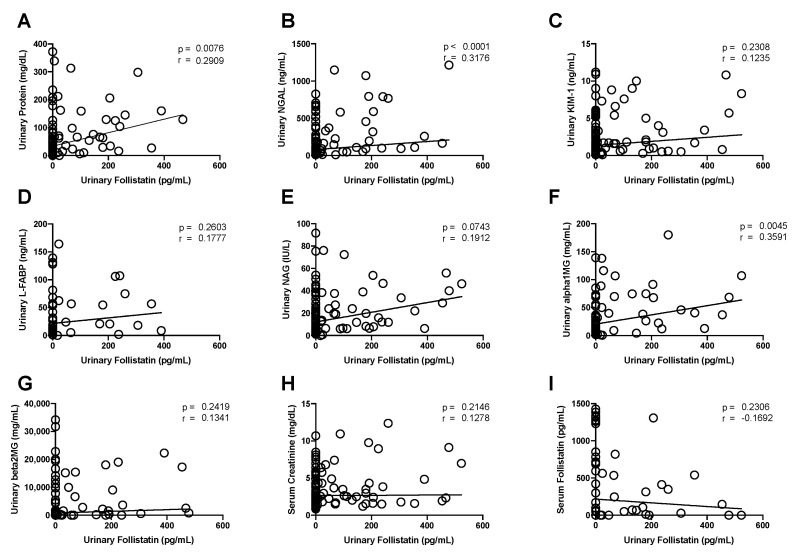
Correlations between Urinary Follistatin and Clinical Parameters. Correlations between urinary follistatin and urinary protein level (**A**), NGAL (**B**), KIM-1 (**C**), L-FABP (**D**), NAG (**E**), α1 microglobulin (**F**), β2 microglobulin (**G**), serum creatinine (**H**), and serum follistatin (**I**) are shown.

We further investigated the association between urinary follistatin levels and various clinical parameters. Urinary follistatin exhibited a significant correlation with urinary protein levels (Figure 3A), while no such correlation was observed with serum creatinine (Figure 3H). Furthermore, a significant correlation was established between urinary follistatin and urinary α1 microglobulin (Figure 3F), whereas no correlation was evident with urinary NAG (Figure 3E) or urinary β2 microglobulin (Figure 3G). In a comparative analysis of urinary follistatin and other acute kidney injury (AKI) biomarkers, we observed a significant correlation between urinary follistatin and urinary NGAL (Figure 3B). However, no significant correlations were observed with urinary KIM-1 (Figure 3C) or L-FABP (Figure 3D). Intriguingly, no substantial correlation was observed between urinary follistatin and serum follistatin levels (Figure 3I), suggesting a renal origin for urinary follistatin.

### 3.6. Time Course of Changes in Urinary Follistatin and Other AKI Biomarkers in an AKI Patient

**Figure 4:**
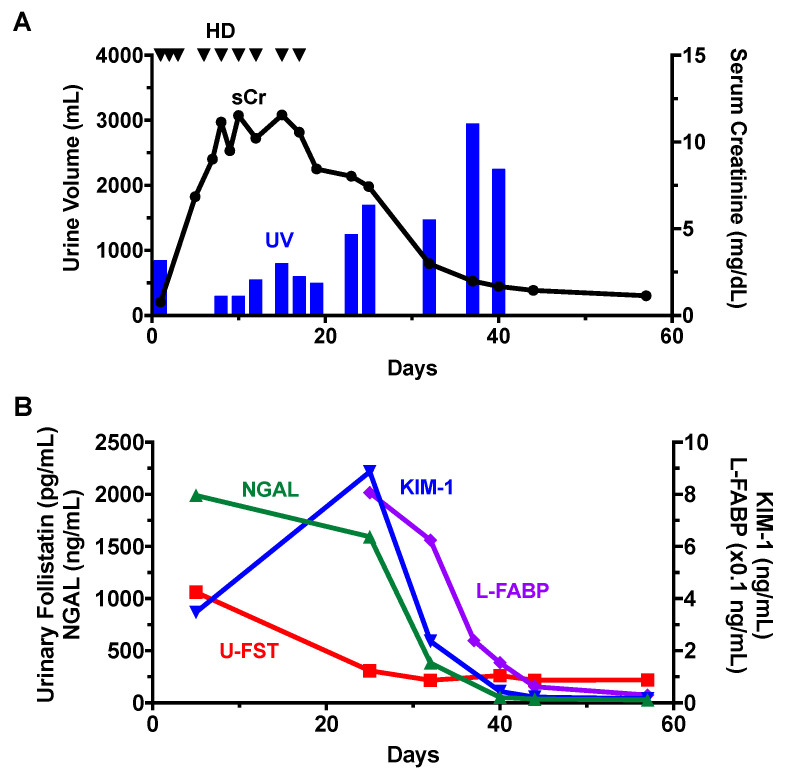
Time Course Changes in Urinary Follistatin and Other AKI Biomarkers. Changes in serum creatinine and urine volume (**A**) and AKI biomarkers (**B**) in a patient with drug-induced AKI are shown. Triangle indicates the timing of hemodialysis (HD).

In the present study, we conducted a comparative analysis of the temporal dynamics of urinary follistatin levels and other AKI biomarkers in a patient experiencing drug-induced AKI. Upon admission, the patient presented with oliguria and required RRT on the following day. RRT was administered for a duration of 18 days, during which the urine volume gradually increased, and renal function subsequently recovered (Figure 4A). Notably, an elevated level of urinary NGAL was observed at the time of AKI diagnosis, which persisted until 7 days post-discontinuation of RRT. Concurrently, an increase in urinary KIM-1 was noted at the time of AKI diagnosis. The peak of urinary KIM-1 occurred at 7 days post-cessation of RRT, followed by a subsequent decline. In contrast, urinary follistatin levels demonstrated a significant elevation at the onset of AKI; however, distinct from NGAL and KIM-1, these levels rapidly diminished before the normalization of serum creatinine (Figure 4B).

### 3.7. Urinary Follistatin in Patients with Different Causes of AKI

**Figure 5:**
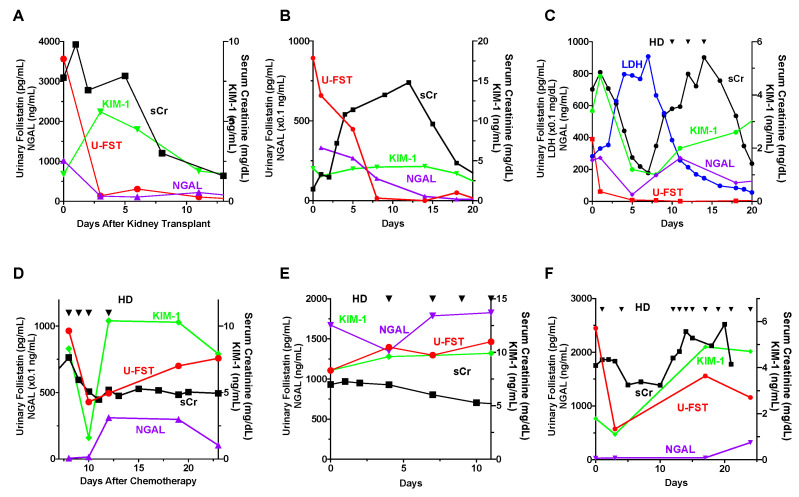
Time Course Changes in Urinary Follistatin in Patients with AKI. (**A**–**F**): Changes in serum creatinine, urinary NGAL, urinary KIM-1, and urinary follistatin levels in patients with various causes of AKI, including cadaver kidney transplantation (**A**), drug-induced nephropathy (**B**), Burkitt lymphoma accompanied by tumor lysis syndrome (**C**), chemotherapy (**D**), contrast-induced nephropathy (**E**), and cholesterol crystal embolism (**F**), are shown. Triangle indicates the timing of hemodialysis (HD).

We examined the temporal dynamics of alterations in serum creatinine, urinary follistatin, and other urinary biomarkers in patients with diverse etiologies of AKI. In the setting of cadaveric kidney transplantation, urinary follistatin levels exhibited an initial elevation immediately post-renal transplantation, followed by a gradual decline to undetectable levels thereafter (Figure 5A). For instances of drug-induced AKI (Figure 5B) or AKI associated with Burkitt lymphoma accompanied by tumor lysis syndrome (Figure 5C), urinary follistatin levels returned to baseline prior to the normalization of serum creatinine levels. Among patients experiencing chemotherapy-induced AKI necessitating RRT, urinary follistatin levels increased promptly after the initiation of chemotherapy, but subsequently exhibited a rapid decline (Figure 5D). Conversely, in cases of AKI progressing to irreversible ESRD due to contrast-induced nephropathy (Figure 5E) or cholesterol crystal embolism (Figure 5F), elevated levels of urinary follistatin persisted until discharge.

## 4. Discussion

As is consistent with previous reports demonstrating the localization of follistatin protein in the renal tubules of normal rats [25,26], we observed that follistatin is localized in the distal tubules of normal human kidneys (Figure 1A–C). Notably, we observed that follistatin is undetectable in the urine of healthy controls, but is markedly elevated in the urine of patients with AKI (Figure 2). Moreover, urinary follistatin levels exhibited a correlation with the severity of AKI (Figure 3). Importantly, no significant correlation was identified between serum follistatin and urinary follistatin (Figure 3I), which suggests that urinary follistatin originates from the kidneys rather than the bloodstream. Collectively, our findings propose urinary follistatin as a novel tubular injury marker with potential utility for monitoring AKI in clinical settings. Although serum follistatin levels have been extensively investigated in various diseases, including muscle physiology [27], metabolic disorders [28,29,30], cancer [31,32,33], and cardiovascular diseases [34,35], to the best of our knowledge, the present study represents the first demonstration that urinary follistatin is present in AKI patients, identifying it as a novel biomarker for detecting renal diseases. This discovery highlights the potential use of follistatin in the assessment of kidney health and the diagnosis of renal disorders.

In the present study, we conducted a comparative analysis of the time course changes in urinary follistatin with several biomarkers. Neutrophil gelatinase-associated lipocalin (NGAL), a highly sensitive early biomarker for kidney injury [10], displayed early elevation in conditions such as AKI, diabetic nephropathy, nephrotic syndrome, and obstructive nephropathy [36]. Urinary follistatin exhibited a significant correlation with urinary NGAL (Figure 3B) and displayed a similar trend to urinary NGAL in many cases (Figure 4 and Figure 5A–C,E), suggesting the potential utility of urinary follistatin as an early diagnostic marker for AKI. In humans, urinary NGAL increased not only in AKI, but also in chronic kidney disease (CKD) [37], and is a marker for delayed graft function following kidney transplantation [38], suggesting that the potential of urinary follistatin as a tubular injury marker is not limited to AKI.

Kidney injury molecule-1 (KIM-1) serves as a valuable biomarker for the definitive diagnosis of AKI [11]. A soluble form of human KIM-1 can be detected in the urine of patients with acute tubular necrosis and serves as a useful biomarker for renal proximal tubule injury facilitating the early diagnosis of the disease and serving as a diagnostic discriminator [11]. Our study revealed that urinary follistatin exhibited a generally analogous pattern to KIM-1, with heightened sensitivity in specific scenarios such as renal transplantation (Figure 5A) and tumor lysis syndrome (Figure 5C). While a strong correlation was observed between urinary follistatin and urinary KIM-1 in a rat model of ischemia-reperfusion injury [25], urinary follistatin did not correlate with KIM-1 in our human study. This discrepancy may arise from differences in the timing of urine collection after AKI, highlighting the importance of considering variations in sample collection timing.

The results of the present study must be viewed in light of several limitations. First, the analysis was conducted on a small number of subjects from a single center, potentially limiting the generalizability of our findings to the broader spectrum of AKI. The absence of consistent data on daily or hourly urine output trends necessitated the definition of AKI solely based on serum creatinine criteria. Secondly, the short follow-up period of AKI patients (median, 38 days) precludes a comprehensive assessment of the impact of urinary follistatin on renal prognosis. Additionally, the variable timing of sample collection after the onset of AKI introduced heterogeneity in our patient cohort, encompassing both the injury and recovery phases. There was significant variation in the number of cases among each etiological group. Furthermore, the lack of investigation into follistatin in the renal tissue of AKI patients leaves unanswered questions regarding its increase in human kidneys with AKI, as observed in rats [25]. The mechanism underlying the appearance of follistatin produced in distal tubules in the urine during AKI remains unclear in our study. To clarify the significance of urinary FST measurement in AKI, it is necessary to confirm the changes in the expression site and amount of FST in the renal tissue of cases with AKI. However, renal biopsy, an invasive examination, is not routinely performed in cases of AKI. In cases of AKI with unknown causes, a renal biopsy is performed to investigate the cause, but in many cases of AKI, the causative factor is known beforehand, so histological evaluation by renal biopsy is not necessary and treatment decisions can be made even without renal biopsy results. Therefore, the number of renal biopsy specimens from cases of AKI is very small. Furthermore, it is difficult to confirm the changes in the expression site and amount of FST associated with AKI by evaluation through renal biopsy. In this study, we observed that the expression site of FST in healthy kidneys is widely distributed from the cortex to the medulla. Since the tissue usually collected in a renal biopsy consists mostly of the cortex, it is not possible to observe the expression of FST in the medulla. One possibility is that the mechanism involves the breakdown of tubular cells, akin to NAG, causing intracellular follistatin to leak into the urine. Additionally, factors such as ischemia and hypoxia associated with AKI may increase the expression and production of follistatin in the tubules, leading to its leakage into the urine. Further study is required in order to address this issue.

The results of the present study highlight a significant increase in urinary follistatin levels in patients with AKI, with this elevation correlating with the severity and prognosis of AKI. The concurrent assessment of urinary follistatin along with other biomarkers holds the potential to facilitate the surveillance of AKI progression and aid in determining the optimal timing for interventions.

## 5. Conclusions

Urinary follistatin may serve as a valuable marker reflecting the severity of acute tubular damage in sepsis-associated AKI and drug-induced AKI.

## Figures and Tables

**Table 1:** Baseline Characteristics of Patients with Acute Kidney Injury (AKI) and Healthy Controls. Data were collected from healthy controls and patients with AKI at the initial visit. Abbreviations: AKI, acute kidney injury; BMI, body mass index; CKD, chronic kidney disease; eGFR, estimated glomerular filtration rate; WBC, white blood cell; CRP, C-reactive protein; NAG, N-acetyl-β-D-glucosaminidase; α1MG, α1 microglobulin; β2MG, β2 microglobulin, KIM-1, kidney injury molecule-1; NGAL, neutrophil gelatinase-associated lipocalin; L-FABP, liver-type fatty acid-binding protein; FENa, fractional excretion of sodium; FEurea, fractional excretion of urea.

	AKI						Healthy Control					
	n = 118						n = 16					P
Age, years, mean ± S.E.	62.2	±		16.6			54.9	±		2.9		0.379
BMI, kg/m2, mean ± S.E.	24.1	±		5.2			23.4	±		0.8		0.596
Male gender, n (%)	80		(	67.8	)		2		(	12.5	)	<0.001
Weight, kg, mean ± S.E.	63.3	±		15.8			60	±		3.2		0.493
												
Complications, n (%)												
Diabetes	37		(	31.4	)		1		(	6.3	)	0.054
Hypertension	78		(	66.1	)		7		(	43.8	)	0.294
Dyslipidemia	43		(	36.4	)		1		(	6.3	)	0.037
Old myocardial infarction	11		(	9.3	)		0		(	0.0	)	0.226
Old cerebral infarction	6		(	5.1	)		0		(	0.0	)	0.401
Angina pectoris	12		(	10.2	)		0		(	0.0	)	0.253
Chronic obstructive pulmonary disease	5		(	4.2	)		0		(	0.0	)	0.401
Liver disease	4		(	3.4	)		0		(	0.0	)	0.455
												
Hematological data, mean ± S.E.												
Na, mEq/L	137	±		5.5			142	±		0.5		0.005
K, mEq/L	4.4	±		0.9			4.2	±		0.1		0.379
Cl, mEq/L	103	±		6.3			106	±		0.6		0.124
BUN, mg/dL	55	±		33.2			14	±		0.8		<0.001
Creatinine, mg/dL	4.52	±		3.55			0.68	±		0.04		<0.001
eGFR, mL/min	19.1	±		16.7			79.9	±		3.6		<0.001
Hemoglobin, g/dL	10.6	±		2.9			13.3	±		0.2		0.015
Platelets, x104/μL	74.8	±		109.3			26.1	±		1.7		0.195
WBC, x103/μL	6.9	±		6.8			5.7	±		0.4		0.462
CRP, mg/dL	7.93	±		9.0			0.24	±		0.1		0.015
												
Urinalysis, mean ± S.E.												
Urinary protein, mg/dL	245.1	±		494.6				-				-
NAG, IU/L	33.6	±		39.1				-				-
α1MG, mg/L	55.4	±		66.7				-				-
β2MG, mg/L	12.5	±		24.2				-				-
NGAL, ng/mL	1206.7	±		2750.9				-				-
KIM-1, ng/mL	3.8	±		7.5				-				-
L-FABP, ng/mL	168.4	±		480.5				-				-
FENa, %	5.0	±		7.4				-				-
FEUrea, %	36.5	±		22.6				-				-

## Data Availability

The datasets used and analyzed in the current study are available from the corresponding author upon reasonable request.
